# An In Vitro Study of Mesiobuccal Root Thickness of Maxillary First Molars

**Published:** 2012-03-01

**Authors:** Nahid Mohammadzadeh Akhlaghi, Yasaman Ravandoust, Mohammad Najafi, Bahareh Dadresanfar

**Affiliations:** 1. Department of Endodontics, Dental Branch, Islamic Azad University, Tehran, Iran; 2. Private Practice, Tehran, Iran

**Keywords:** Instrumentation, Profile, Root Canal Preparation, Stainless Steel, Thickness

## Abstract

**Introduction:**

Understanding the internal anatomy of root canal system can significantly influence outcomes of root canal treatment. The aim of this in vitro study was to measure the thickness of mesiobuccal root at different levels in maxillary first molars.

**Materials and Methods:**

In this cross-sectional study, forty extracted human maxillary first molars were radiographed; accordingly, the mesial and distal root thicknesses of mesiobuccal (MB) roots were measured at four parallel horizontal levels. The samples were sectioned at the measured levels and then sections were scanned and saved in the computer. Buccal (B), Palatal (P), Mesial (M) and Distal (D) aspects of root thicknesses in single-canalled roots were measured. In two–canalled mesiobuccal roots, Distobuccal (DB) and Distopalatal (DP) aspects were evaluated alongside other measurements. Average radicular thickness in each aspect and each level was compared using ANOVA and t-test.

**Results:**

A total of 25 had two canals and 15 had one canal in MB root. In single-canalled roots M and D aspects were the thinnest whereas in two-canalled samples, the thicknesses of DP and DB aspects were significantly less than others (P<0.001). The B and P had the greatest thicknesses in all the samples.

**Conclusion:**

The results showed that special attention should be paid to "danger zone” areas of mesiobuccal maxillary first molar roots in order to avoid technical mishaps.

## Introduction

Thorough knowledge of root canal anatomy is essential for successful endodontic therapy. Mesiobuccal (MB) root of maxillary molars presents variable buccolingual dimension, and in most cases it encloses two canals [1].

Verma and Love [2] have reported a second mesiobuccal canal in 90 percent of the examined roots. This anatomy results in concavity of mesial and distal surfaces in roots known as danger zones. It is obvious that the thickness of these surfaces has direct correlation with the outcome of root canal treatment and the successive restorative procedures [3][4].

In a study performed by Garala et al. the importance of pre-operative canal wall thickness as the most significant factor determining the outcome of canal preparation has been emphasized [4]. Also it has been shown that the remaining thickness of walls after preparation might be the most important iatrogenic factor that correlates with incoming fracture resistance [5]. There are several studies regarding root canal morphology in mesiobuccal root of maxillary molars [1][2][6][7], however, very few information about the root thickness of different walls of this root is present [8].

The purpose of this study was to evaluate cement/dentin thickness of MB root of maxillary first molars at four horizontal levels by means of radiography and sectioning.

## Materials and Methods

**Figure 1:**
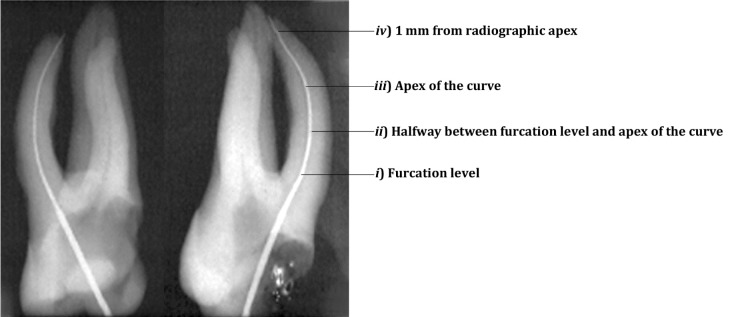
Radiographic location of the three measured levels of mesiobuccal root thickness in maxillary first molar

In this in vitro study, 40 mature extracted human maxillary first molars from individuals aged range 26-50 were collected by random sampling. The teeth were placed in 5.25% NaOCl for one hour, immersed in saline and then the surfaces of the roots were cleaned ultrasonically. Access cavities were provided and#10 K-file (Maillefer, Dentsply, Ballaigues, Switzerland) was introduced to MB1 canal and the teeth were buccolingually radiographed. Then the X-rays were scanned (Scanjet 44 Foc, Hewlett-Packard, Germany) and canal curvature were determined using AutoCAD 2002 according to Schneider's method [9]. Anatomic root thicknesses were measured; radiographic root thicknesses of mesial and distal aspects were measured using Adobe Photoshop (0.01 mm accuracy) at four parallel horizontal levels (Figure 1).

i. Furcation level

ii. Halfway between furcation level and apex of the root curve

iii. Apex of the curvature: the intersection of coronal and apical long axis according to Calberson et al.[10]

iv. 1 mm above the radiographic apex

The crowns were then cut off and the mesiobuccal (MB) roots were colored by eosin, so the canal outline could be better distinguished. The roots were then embedded in acrylic blocks.

The blocks were then horizontally sectioned by D&Z disk (0.2 mm thickness) (Drendel, Zweiling, Berlin, Germany) at the four levels described before. The sections were scanned and saved in computer with 1200-2400 pixel clearance and observed under ×20 magnification with 0.01 mm accuracy. For each section, buccolingual and mesiodistal central axes were drawn. Mesial root thickness was determined by the distance between external limit of mesial root surface and mesial border of the canal. Distal (D), palatal (P), and buccal (B) aspects of MB root thicknesses were measured respectively in single-canalled roots. In two-canalled roots minimum distance between MB1 canal and distal limit of the section was considered Distopalatal (DP) areas and minimum distance between MB2 canal and the corresponding distal side of the section was considered as Distobuccal (DB) areas Anatomic and radiographic root thicknesses were analyzed and compared by parametric tests like repeated measured ANOVA, post hoc Tukey and t-test.

## Results

Of forty maxillary first molars included in this study, 62.5% (25 teeth) had two canals and 37.5% (15 teeth) had one canal in MB root. Average canal curvature was 23˚. Table 1-2 show MB root thickness of maxillary first molars (single- and double-canalled roots).

**Table 1:** Mean (Standard Division) of root thickness (mm) of double-canalled maxillary first molars (N=25)

**Level**	**MB1**				**MB2**			
	**B**	**M**	**D**	**DP**	**P**	**M**	**D**	**DB**
**1**	1.75 (0.31)	1.37 (0.21)	1.32 (0.21)	1.18 (0.18)	1.57 (0.42)	1.03 (0.30)	0.89 (0.17)	0.81 (0.15)
**2**	1.50 (0.31)	1.23 (0.28)	1.15 (0.26)	1.06 (0.19)	1.19 (0.39)	0.85 (0.18)	0.78 (0.18)	0.72 (0.19)
**3**	1.30 (0.33)	1.1 (0.20)	1.1 (0.20)	0.97 (0.20)	1.0 (0.43	0.78 (0.22)	0.74 (0.15)	0.69 (0.13)
**4**	0.97 (0.32)	0.87 (0.24)	0.80 (0.21)	0.76 (0.19)	0.75 (0.19)	0.61 (0.23)	0.56 (0.13)	0.51 (0.12)

**Table 2:** Mean (Standard Division) of mesiobuccal root thickness (mm) of single-canalled maxillary first molars (N=15)

**Level **	**B**	**M**	**D**	**p**
**1**	2.0 (0.27)	1.23 (0.31)	0.98 (0.29)	1.85 (0.24)
**2**	1.84 (0.23)	1.04 (0.20)	0.89 (0.23)	1.58 (0.26)
**3**	1.60 (0.33)	0.95 (0.17)	0.84 (0.18)	1.39 (0.33)
**4**	0.93 (0.22)	0.73 (0.20)	0.72 (0.15)	0.85 (0.18)

Overall evaluation of cross-sections showed that in single-canalled roots, B aspects were the thickest (1.39±0.59 mm) and D aspects were the thinnest (0.76±0.26 mm). On the other hand in two-canalled roots B aspects of MB1 canals (1.51±0.39 mm) and P aspects of MB2 canals (1.27±0.42 mm) had the greatest thicknesses. Whereas, DP aspects (1.05±0.22 mm) of MB1 canals and DB aspects (0.73±0.17 mm) of MB2 canals had the least amount of cement/dentin thicknesses (P<0.001) (Table 3).

**Table 3:** Mean (Standard Division) of overall average of mesiobuccal root thickness (mm) of maxillary first molars (N=40)

**Level **	**MB1**	**MB2**	**Single Canalled Roots**
**1**	1.40 (0.31)	1.07 (0.41)	1.51 (0.51)
**2**	1.23 (0.30)	0.89 (0.31)	1.34 (0.45)
**3**	1.11 (0.27)	0.82 (0.30)	1.19 (0.41)
**4**	0.85 (0.20)	0.61 (0.19)	0.80 (0.22)

Single-canalled roots had a significantly higher average of overall root thicknesses (1.21±0.51 mm) compare to double-canalled roots (1.08±0.39 mm) (t-test, P<0.001) (Table 4). Nevertheless, average root thicknesses of proximal aspects were lower in single-canalled roots. In all the samples average root thickness of M aspects were significantly higher than D aspects.

**Table 4:** Mean (Standard Division) of overall average of level thickness (mm) (N=40)

**MB1**				**MB2**				**Single Canalled Roots**			
**B**	**M**	**D**	**DP**	**P**	**M**	**D**	**DP**	**B**	**M**	**D**	**P**
1.51 (0.39)	1.23 (0.26)	1.17 (0.26)	1.05 (0.22)	1.27 (0.48)	0.89 (0.28)	0.79 (0.19)	0.73 (0.17)	1.39 (0.59)	0.90 (0.30)	0.76 (0.26)	1.19 (0.57)

Radiographic evaluations indicated that the thicknesses of M aspects (1.21±0.41 mm) were significantly higher than D aspects (1.01±0.40mm). Also in comparison to anatomic evaluation a 19% higher mean value for D aspect and 16% for M one was noted (P<0.05).

## Discussion

Aside from an adequate procedural concept for a successful endodontic treatment, precise knowledge of tooth anatomy is the fundamental factor in visualizing the final outcome of treatment. The maxillary first molar was the subject of our study due to little information found in the literature concerning the thickness of the mesiobuccal root.

One of the notable aspects of this study was to evaluate the thicknesses of distopalatal and distobuccal surfaces of MB1 and MB2 canals respectively. These concave areas, naturally present in distal aspect of mesiobuccal root of maxillary molars, as shown in our study have special clinical importance and can indeed be considered as danger zones. Furthermore as root thickness of these danger zones decreased from coronal to apical, the differences became significant.

Berruti and Fedon [3] and Akhlaghi et al. [11] in different studies confirmed the existence of such concavities on distal surface of mesial root of mandibular first molars. Their study, like ours, showed a constant decrease in amount of cement/dentin towards apical sections.

The averages reported by Degerness and Bowles [8] for the thickness of MB root of maxillary molars are similar to our findings and very few differences could be due to ethnic backgrounds, age and gender of the studied samples.

In a similar study by Hűbscher et al., canal shape analysis was done on maxillary molars by micro-computed tomography to compare pre and post-operative geometrical changes in prepared canals [12]. They evaluated the volume and surface area changes and not specifically the amount of root cement/dentin. On the other hand concave areas in mesiobuccal root were not included in that study and only 11 teeth were studied which does not seem to be adequate size of sample.

In our study buccal and palatal surfaces of mesiobuccal root in both single and two-canalled roots had the highest thicknesses which is similar to the other reports in this field for anterior teeth and premolars [8][13].

Also in comparing single and double-canalled roots, although the overall average of root thickness of various surfaces was higher in single-canalled roots, the average of mesial and distal thicknesses were lower which is the result of ribbon shaped appearance of single canals reported previously by Hűbscher et al. as well [12]. Also in a study of maxillary first premolars Raiden et al. demonstrated an hour-glass shaped section for the single-canalled samples [14]. In a recent study by Degerness and Bowles [15] it has been suggested that the danger zone of maxillary molars is located at a level where the root joins the crown of the tooth. Therefore it is reasonably recommended to avoid weakening of distal surfaces in mesiobuccal root of maxillary molars regardless of number of canals in the root.

According to some studies [4][16][17] dentin removal in more coronal sections of the canal is mostly toward distal aspects, whereas in apical parts it tends to happen in mesial surfaces. Our results showed danger zones locating in distal aspect of mesiobuccal root between furcation level and apex of the curve. In a study by Shahriari et al. it was emphasized that stainless steel instruments tend to remove more dentin from danger zone area and ProFile rotary instrumentation conserves more root dentin [18]. Thus it's advisable to pay special attention to these areas specially during pre-flaring.

Comparison of radiographic and anatomic measurements showed a higher mean value of 19% for distal and 16% for mesial surfaces were noticed which is close to the result of Berutti and Fedon who reported 20% higher mean value in mandibular molars [3]. Raiden et al. reported that radiographic method does not seem a proper way for evaluation of root thickness [19]. Clinical relevance of this discrepancy would help the clinicians to keep in mind that real root thicknesses are always less than what appears in the pre-operative radiographs.

Our findings from the apical level showed that all surfaces had less than one millimeter thicknesses. This encourages further studies to create a balance between disinfection of the canal due to apical instrumentation and maintenance of the initial canal configuration.

## Conclusion

Based on the findings of the present study, special attention should be paid to the preparation of apical area of mesiobuccal root in maxillary molars due to thicknesses of less than 1mm. Also, it should be considered that dental radiographs cannot reveal these areas precisely.
